# Crystal Structures of Two Transcriptional Regulators from *Bacillus cereus* Define the Conserved Structural Features of a PadR Subfamily

**DOI:** 10.1371/journal.pone.0048015

**Published:** 2012-11-26

**Authors:** Guntur Fibriansah, Ákos T. Kovács, Trijntje J. Pool, Mirjam Boonstra, Oscar P. Kuipers, Andy-Mark W. H. Thunnissen

**Affiliations:** 1 Laboratory of Biophysical Chemistry, Groningen Biomolecular Sciences and Biotechnology Institute, University of Groningen, Groningen, The Netherlands; 2 Department of Genetics, Groningen Biomolecular Sciences and Biotechnology Institute, University of Groningen, Groningen, The Netherlands; Centre National de la Recherche Scientifique, Aix-Marseille Université, France

## Abstract

PadR-like transcriptional regulators form a structurally-related family of proteins that control the expression of genes associated with detoxification, virulence and multi-drug resistance in bacteria. Only a few members of this family have been studied by genetic, biochemical and biophysical methods, and their structure/function relationships are still largely undefined. Here, we report the crystal structures of two PadR-like proteins from *Bacillus cereus*, which we named *bc*PadR1 and *bc*PadR2 (products of gene loci BC4206 and BCE3449 in strains ATCC 14579 and ATCC 10987, respectively). BC4206, together with its neighboring gene BC4207, was previously shown to become significantly upregulated in presence of the bacteriocin AS-48. DNA mobility shift assays reveal that *bc*PadR1 binds to a 250 bp intergenic region containing the putative BC4206–BC4207 promoter sequence, while *in-situ* expression of *bc*PadR1 decreases bacteriocin tolerance, together suggesting a role for *bc*PadR1 as repressor of BC4206–BC4207 transcription. The function of *bc*PadR2 (48% identical in sequence to *bc*PadR1) is unknown, but the location of its gene just upstream from genes encoding a putative antibiotic ABC efflux pump, suggests a role in regulating antibiotic resistance. The *bc*PadR proteins are structurally similar to LmrR, a PadR-like transcription regulator in *Lactococcus lactis* that controls expression of a multidrug ABC transporter via a mechanism of multidrug binding and induction. Together these proteins define a subfamily of conserved, relatively small PadR proteins characterized by a single C-terminal helix for dimerization. Unlike LmrR, *bc*PadR1 and *bc*PadR2 lack a central pore for ligand binding, making it unclear whether the transcriptional regulatory roles of *bc*PadR1 and *bc*PadR2 involve direct ligand recognition and induction.

## Introduction

Members of the PadR-like protein family (accession no. Pfam PF03551) form a large and widespread group of bacterial transcription factors that regulate diverse processes including multi-drug resistance, virulence and detoxification. They are named after the phenolic acid decarboxylation repressor, which is a transcription factor that represses genes associated with the phenolic acid stress response in gram-positive bacteria such as *Bacillus subtilis*, *Pediococcus pentosaceus*, and *Lactobacillus plantarum* [1], [2]. Besides the founding member, only a few of the PadR-like proteins have been or are currently under investigation. These include AphA, which activates virulence gene expression in *Vibrio cholerae* [3], LmrR and LadR, which are repressors of genes encoding a multi-drug resistance pump in *Lactococcus lactis* and *Listeria monocytogenesis*, respectively [4], [5], and Pex, a regulator of circadian rhythms in *Synechococcus elongates* [6]. Crystal structures have been reported of AphA, LmrR and Pex, confirming that these proteins share a common fold consisting of two domains: a highly conserved N-terminal winged helix-turn-helix (wHTH) domain of approximately 80–90 amino acid residues, involved in DNA-binding, and a variable C-terminal domain of one or more α-helices, involved in dimerization [7]–[9]. PadR-like proteins are related to members of the multiple antibiotic resistance regulator (MarR) protein family, which have similar wHTH DNA-binding domains but significantly larger and distinct C-terminal dimerization domains [10]. The size of the C-terminal domain is also a major discriminator among different PadR-like proteins, which have been classified into two distinct subfamilies [4]. PadR-like proteins of subfamily 1 (hereafter abbreviated as PadR-s1, approximately 180 amino acids), which include AphA, PadR and LadR, have relatively large C-terminal domains of about 80–90 amino acids containing multiple α-helices, while subfamily 2 PadR-like proteins (PadR-s2, approximately 110 amino acids), which includes LmrR, have significantly smaller C-terminal domains of about 20–30 amino acids forming a single α-helix.

Currently, the Protein Data Bank contains structures of only a few PadR-s2 proteins. Among these proteins, LmrR is the only one that has been studied both structurally and functionally: the structures of the other proteins were solved by structural genomics centers without a published record of their functional properties. LmrR regulates the expression of LmrCD, a major multidrug ABC efflux pump of *L. lactis* [11], via a mechanism involving multidrug binding and induction [5], [9]. Forming a negative feedback loop, LmrR also regulates transcription of its own gene, which is situated just upstream from the *lmrCD* genes, [12]. Crystal structures of LmrR in complex with different ligands revealed that LmrR contains an unusual multidrug binding site within a large central hydrophobic pore at its dimer interface [9]. Multidrug recognition is dominated by aromatic stacking interactions of the flat heterocyclic cores of the ligands with opposing indole groups of two dimer-related tryptophan residues (W96/W96′), the prime indicating the dimer-related subunit). Interestingly, the drug-binding tryptophan residue in LmrR, which is located in the C-terminal helix, is conserved in other PadR-s2 proteins, including those with known structure. However, in these latter proteins the equivalent tryptophans are buried in a completely closed dimer interface, making it highly unlikely that they have a similar ligand-binding role [9]. Unfortunately, assessment of the significance of the structural differences between LmrR and the other PadR-s2 proteins is prohibited by the lack of complete functional data.

To investigate whether LmrR is an exceptional member of the PadR-s2 family or whether its structural ligand-binding features are preserved in other members, requires structure/function analysis of LmrR homologs that function in multidrug or antibiotic resistance. Such a homolog of LmrR was recently identified in *Bacillus cereus* as the product of gene locus BC4206. Together with its neighboring gene BC4207, which encodes a putative membrane protein, BC4206 was shown to become highly upregulated if *B. cereus* is treated with low concentrations of enterocin AS-48, a broad-spectrum antimicrobial cyclic peptide produced by *Enterococcus faecalis* S-48 [13], [14]. Here we report the crystal structure of the BC4206 gene product from *B. cereus* strain ATCC 14579, a subfamily-2 PadR-like protein which we named *bc*PadR1. In addition, we report the crystal structure of a *bc*PadR1 homolog from *B. cereus* strain ATCC 10987, the product of gene locus BCE3449, which we named *bc*PadR2. This latter protein was selected for structural characterization because its gene is positioned in the vicinity of two genes encoding a putative antibiotic ABC efflux pump (gene loci BCE3447 and BCE3446). The proximity of these genes, similar as in the *lmrR*-*lmrCD* locus, could indicate that *bc*PadR2 regulates the expression of the ABC efflux pump, perhaps following a similar ligand binding and induction mechanism as LmrR. Preliminary functional analysis, combined with our structural analysis and comparison with related proteins, reveal the roles of various conserved residues in the two subfamily-2 PadR-like proteins from *B. cereus*. Based on our results, we discuss possibilities for ligand-induced regulation of these proteins.

## Materials and Methods

### Cloning

The open reading frames encoding *bc*PadR1 and *bc*PadR2 were amplified by PCR from the genomic DNA of *Bacillus cereus* strains ATCC 14579 and ATCC 10987, respectively, and cloned in the vector pNSC8048 [15]. Plasmid pNSC8048 carries the *nisA* promoter, chloramphenicol selectable marker and a C-terminal strep-tag (SRWSHPQFEK). The forward and reverse primers for *bcpadR1* (locus BC4206) were 5′-CGA**CCATGG**GGCACAGCCAAATGTTAAAAGGTGTA-3′ and 5′- GCT**TCTAGA**CTCCCCCTGTAATAAGTTATT-3′, respectively, while forward and reverse primes for *bcpadR2* (locus BCE3449) were 5′-CGA**CCATGG**AAAATTTAACTGAAATGCTGAAAG and 5′-GCT**TCTAGA**GTTTGACTTCAAGACGTTAAT-3′, respectively. Those primers were designed to create NcoI/XbaI restriction sites of the resulting PCR products (showed as bold and underlined letters). The PCR fragments and pNSC8048 vector were digested with NcoI and XbaI restriction enzymes and the vector was dephosphorylated further with calf intestine alkaline phosphatase (CIAP, Fermentas). Ligation was performed using T4 ligase (Roche) to yield pNSC-*padR*1 and pNSC-*padR*2. DNA sequences of the plasmids were all verified by sequencing (Macrogen, The Netherlands).

### Expression and purification

To express *bc*PadR1 and *bc*PadR2, plasmids pNSC-*padR*1 and pNSC-*padR*2 were transformed into *L. lactis* NZ9000 cells, which is a derivative of strain MG1363 carrying the *nisR*/*nisK* genes crucial for the induction and overexpression of the target protein under the nisin inducible system. The transformed *L. lactis* NZ9000 cells were grown in 100 ml of rich medium (M17, 0.5% glucose and 5 µg/mL chloramphenicol). After overnight growth at 30°C, the culture was transferred into 2 L of fresh media and grown also at 30°C. Overexpression was induced with 5 ng/mL nisin when cells were in the mid log phase (OD_600_ of 0.6–0.8) and growth was continued for two hours. The cells were harvested by centrifugation (8000 rpm for 5 min at 4°C; JLA 10.500 rotor, Beckman), washed with 50 mM Tris-HCl, pH 7.4, and re-centrifuged (8000 rpm for 5 min at 4°C; 5810R rotor, Eppendorf). The pellets were stored at −20°C until further use.

For purification, the cell pellets were resuspended in lysis buffer, containing 100 mM Tris-HCl, pH 8.0, 150 mM NaCl, 1 mM EDTA, supplemented with 10 mg/ml lysozyme and a tablet of a Complete Protease Inhibitor cocktail (Roche). After 1 hour incubation at 30°C, MgSO_4_ (10 mM) and DNAseI (10 µg/mL) were added into the suspension and incubation was continued for another 5 min. The cells were disrupted by sonication and remaining cell debris was removed by centrifugation (12.000 rpm for 10 min at 4°C; 5810R rotor, Eppendorf). *Bc*PadR1 or *bc*PadR2 were purified from the supernatant using Streptactin Sepharose column chromatography, according to the protocol provided by the manufacturer (IBA Biotagnology GmbH). The protein-containing elution fractions (in 100 mM Tris-HCl, pH 8.0, 150 mM NaCl, 1 mM EDTA, 2.5 mM desthiobiotin) were diluted to 50 mM NaCl with buffer A (20 mM Tris-HCl, pH 8.0, 1 mM EDTA and 1 mM dithithreitol) and then applied to a heparin column (5 ml Heparin FastFlow, GE Healthcare), connected to an ÄKTA Explorer FPLC system (GE Healthcare), to remove bound DNA from the protein. The retained protein was eluted by applying a linear gradient (2.5–100% in 15 column volumes) of 2 M NaCl in buffer A. The fractions containing *bc*PadR1 or *bc*PadR2 were pooled and concentrated to ∼8 mg/ml in buffer containing 10 mM Tris-HCl, pH 8.0, 250 mM NaCl, 1 mM EDTA and 1 mM DTT using an Amicon Ultra 3 kDa cut-off concentrator (Millipore). The protein purity was confirmed by silver-stained SDS PAGE.

### Expression of *bc*PadR1 in *B. cereus* and determination of antimicrobial activity of AS-48

Plasmid pNSC-*padR1* was electroporated into *B. cereus* ATCC 14579 harboring the *nisRK* genes on plasmid pNZ9530 [16] and expression was induced with 20 ng/mL of nisin. The antimicrobial activity of AS-48 was determined as the minimal inhibitory concentration (MIC) value against *B. cereus* following previous practice [17]. Bacterial growth was followed in the presence of various concentrations of AS-48 and monitored every 15 minutes using a TECAN GENios Absorbance Reader (TECAN). Without AS-48 addition, the final OD_600 nm_ value (at the end of the exponential phase) for the *B. cereus* culture was about 1.1±0.1. An inhibition curve was made by plotting OD_600 nm_ at the end of incubation versus AS-48 concentration. The minimal inhibitory concentration value was determined from the inhibition curve by interpolation. The lowest concentration of AS-48 at which less than 1% of the total increase in the OD_600_, measured in the absence of AS-48, had occurred, was taken as the MIC value.

### Electrophoretic mobility shift assay

Electrophoretic mobility shift assays (EMSAs) were carried out essentially as described by Susanna *et al* [18]. The promoter regions of *B. cereus* BC4206 and BC4029 genes were obtained by PCR with oligos (for BC4206, 5′-TTCAAGTGCCTTTGGTTC-3′ and 5′-GATGCAACCTTCTAATAC-3′; for BC4029 5′-TTTGCACGTTCTTCAAGC-3′ and 5′-CTTTCAGCATTTGACTTG-3′) and the resulting fragments (250 bp and 347 bp for BC4206 and BC4029, respectively) were end-labeled with [γ-33P]ATP using T4 polynucleotide kinase (Roche Nederland B.V., The Netherlands). Purified *bc*PadR1 protein and its DNA probe were premixed on ice in binding buffer (20 mM Tris HCl (pH 8.0), 5 mM MgCl_2_, 100 mM KCl, 0.5 mM dithiotreitol, 0.05 mg/ml poly(dI-dC), 0.05 mg/ml bovine serum albumin, and 8.7% glycerol) [18]. Reaction mixtures contained poly(dI-dC) that is known to eliminate non-specific DNA binding. Samples were incubated at 30°C, and were loaded on a 6% polyacrylamide gel after 20 min incubation. Gels were run in 1× TBE buffer (0.089 mM Tris, 0.089 mM boric acid, 0.022 mM EDTA) at 90 V for 60 minutes, dried in a vacuum dryer and autoradiographed using phosphoscreens and a Cyclone PhosphorImager (Packard Instruments, Meridien, CT).

#### Gel filtration

Analytical gel filtration was performed with a Superdex 200 PC 3.2/30 gel filtration column (GE Healthcare), mounted on a SMART-system (GE Healthcare). The running buffer contained 20 mM Tris-HCl, pH 8.0, 200 mM NaCl, 1 mM EDTA and 1 mM dithithreitol. The injected protein samples volumes were 20 µl. As molecular weight markers a mixture of five proteins was used: aldolase −158 kDa, bovine serum albumin −67 kDa, ovalbumin −43 kDa, chymotrypsin −25 kDa, and ribonuclease −13.7 kDa.

#### Crystallization

Prior to crystallization trials, *bc*PadR1 was subjected to Thermofluor-based stability optimization experiments [19] that were performed on a thermal cycler (myIQ, Bio-Rad) using Sypro-Orange dye (Invitrogen). The results revealed that *bc*PadR1 was most stable in a buffer containing 20 mM MES pH 6.0, 250 mM NaCl, 1 mM EDTA and 1 mM DTT. Accordingly, both *bc*PadR1 and *bc*PadR2 were buffer exchanged to the aforementioned buffer using a PD-10 desalting column (GE Healthcare). The proteins were concentrated further to ∼10 mg/ml and either used immediately for crystallization or stored at −80°C.

Initial crystallization trials were set up as vapour-diffusion sitting drops at 298 K using various commercially available screens [i.e., JCSG+ (Molecular Dimensions), Wizard Screen (Emerald Biosystem), Structure Screen (Emerald Biosystem) and Cryo Screen (Hampton Research)] and a Mosquito crystallization robot (TTP LabTech) for drop dispensing. Droplets were composed of 0.2 µl protein stock solution and 0.2 µl crystallization solution and were equilibrated against 60 µl of the crystallization solution. Trigonal pyramid crystals of *bc*PadR1 were obtained from JCSG+ Screen condition no. 45, containing 0.17 M ammonium sulfate, 25.5% (w/v) PEG 4000 and 15% (v/v) glycerol. These crystals were used immediately for data collection without further optimization. For *bc*PadR2, similar crystals grew from JCSG+ condition no. 60 (0.1 M imidazole, pH 8.0, and 10% (w/v) PEG 8000) which was optimized manually using the hanging-drop vapour diffusion method. The final crystallization solution contained 0.1 M imidazole pH 8.0 and 9% (w/v) PEG 8000.

#### Data collection and structure determination

Prior to diffraction data collection, a single *bc*PadR1 crystal with dimensions of ∼160 µm×160 µm×180 µm was transferred to a cryo-protectant solution containing 0.17 M ammonium sulfate, 27% (w/v) PEG 4000 and 20% (v/v) glycerol, and subsequently dipped into liquid nitrogen. A similar procedure was applied to a *bc*PadR2 crystal (∼100 µm×100 µm×160 µm) with 0.1 M imidazole, pH 8.0, 10% (w/v) PEG 8000 and 20% (v/v) ethylene glycol as cryo-protectant. Diffraction data up to 2.5 Å and 2.2 Å resolution for *bc*PadR1 and *bc*PadR2, respectively, were collected at 100 K using beam line ID14-2 at the ESRF, Grenoble, equipped with an ADSC Quantum 4 detector (see Table 1 for data collection statistics). The two data sets were indexed and integrated in space group *P*4 with the program XDS [20], reindexed to space group *P*422 and scaled and merged with the program Pointless and Scala [21], respectively, from the CCP4 software package [22]. Data collection statistics are shown in Table 1.

**Table 1:** Data collection and refinement.

	*bc*PadR1	*bc*PadR2
***Data collection***		
Wavelength (Å)	0.9330	0.9330
Space group	*P*4_1_2_1_2	*P*4_3_2_1_2
Unit cell dimensions (Å)	a = b = 73.5, c = 84.1	a = b = 43.9, c = 120.1
Resolution (Å)*	44–2.5 (2.64–2.5)	44–2.2 (2.32–2.2)
Total observations	65429	49981
Unique reflections	8474	6500
<I/σ>*	20.6 (2.9)	27.9 (3.3)
Completeness (%)*	100 (100)	99.7 (98.1)
R_merge_ *	0.074 (0.611)	0.042 (0.511)
***Refinement***		
Resolution (Å)	37–2.5	41–2.2
R-factor/R_free_ (%)§	20.2/22.6	22.3/27.7
Number of atoms		
Non-H protein	826	825
Water	17	17
Ligand	28 (glycerol, sulfate)	0
Average B (Å^2^)	58.3	67.8
RMSD		
Bond lengths (Å)	0.003	0.010
Bond angles (°)	0.702	1.010
Ramachandran plot		
% most favoured	98.0	97.9
% disallowed	0.0	0.0

* Values in parentheses refer to the highest resolution shell.

§ The R-factor is calculated for all measured reflections in the specified resolution range, while the R_free_ is calculated for reflections belonging to a random test set not used in refinement (10% of the data).

The structures of *bc*PadR1 and *bc*PadR2 were solved by molecular replacement using the program Balbes [23] running on the YSBL web server (http://www.ysbl.york.ac.uk/YSBLPrograms/). The best solutions for the two proteins were found using the structure of a transcriptional regulator from *Enterococcus faecalis* V583 (PDB entry 3hhh) as a search model and by changing the space groups to *P*4_1_2_1_2 and *P*4_3_2_1_2 for *bc*PadR1 and *bc*PadR2, respectively. The solutions were subjected to automated model building using ARP/wARP [24] via the ARP/wARP web service (http://cluster.embl-hamburg.de/ARPwARP/remote-http.html), and completed by applying several cycles of manual model building using COOT [25] and restrained-refinement using Phenix.refine [26]. TLS (Translation/Libration/Screw) parameters [27] were included in the final rounds of refinement, defining the HTH-domain and C-terminal helix as two separate TLS groups. The final models of *bc*PadR1 and *bc*PadR2 contain 103 residues (amino acids 3–105) and 99 residues (amino acids 3–66 and 72–106), respectively. The geometry of the models was validated by Molprobity [28].

#### Structure and sequence analysis

Structure and sequence analysis was carried out using the following software: SuperPose [29], for superimposition and calculation of root mean square deviations (RMSDs); ESPript [30] and Jalview (http://www.jalview.org) for preparation of sequence alignments; and Pymol (Schrödinger, LLC) for the depiction of structures and preparation of figures.

### Accession numbers

Structure factors and the coordinates of the final models of *bc*PadR1 and *bc*PadR2 have been deposited in the Protein Data Bank (http://www.rcsb.org) with accession numbers 4ESB and 4ESF, respectively.

## Results

### Gene targets of bcPadR1 and bcPadR2

**Figure 1:**
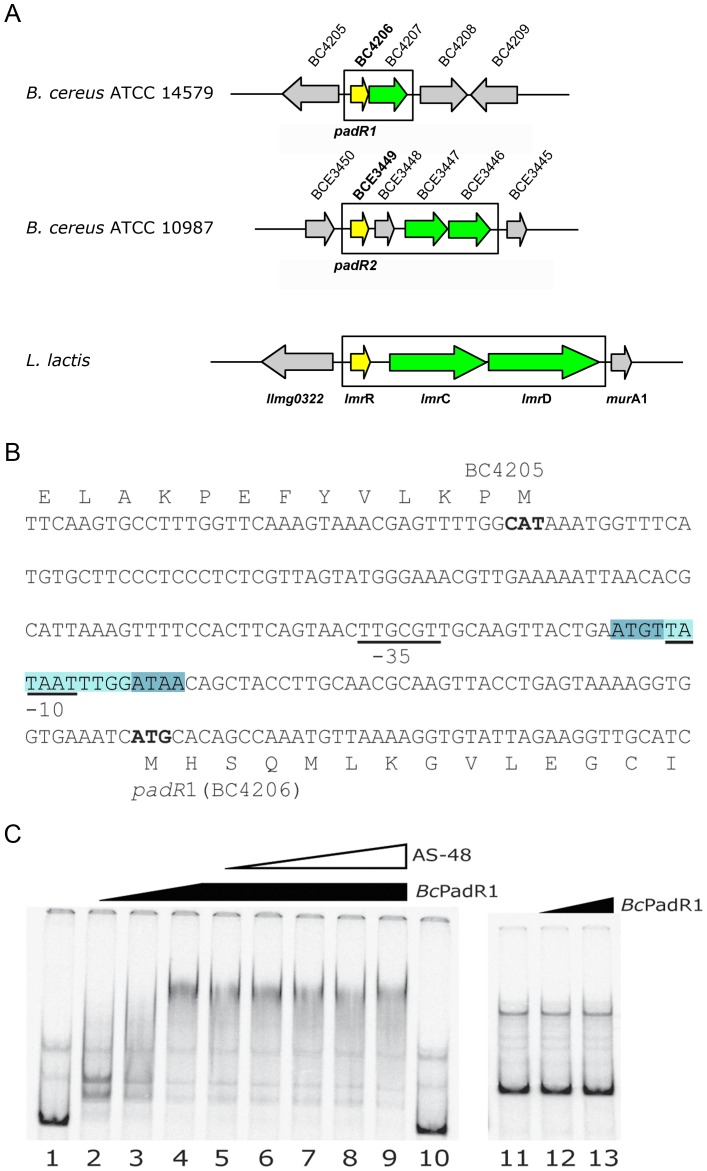
Genomic neighborhood of the *bc*PadR1 and *bc*Padr2 encoding genes and analysis of promoter binding. (A) Organization of the BC4206(*bc*PadR1)-BC4207 operon of *B. cereus* ATCC 14579, the putative BCE3449(*bc*PadR2)-BCE3448-BCE3447-BCE3446 regulon of *B. cereus* ATCC 10987 and the *lmrR*-*lmrCD* regulon of *L. lactis*. The relative scale of the genes and intergenic regions is proportional to nucleotide length. Boxes indicate the operon/regulon boundaries. The PadR-encoding genes are colored in yellow, while their (putative) target genes, encoding resistance-associated membrane proteins, are in green. (B) The sequence of the intergenic region between BC4205 and BC4206 that is used for the EMSA experiments. Putative -35 and -10 promoter sequences of BC4206 are underlined, the start codons of BC4205 and BC4206 are highlighted in bold, and the amino acids of the coded proteins are indicated above the DNA sequence. The putative binding site of *bc*PadR1 (homologous to the canonical ATGT/ACAT inverted sequence motif) is highlighted in blue. (C) EMSA experiments with *bc*PadR1. DNA fragments encompassing the promoter regions of BC4206 (lanes 1–10) and BC4029 (lanes 11–13, negative control) were prepared by PCR and end labeled with ^33^P. DNA binding was assayed as described in the Materials and Methods. Lanes 1, 10 and 11 contain the free DNA probe. Samples run on lanes 2 and 12 contain 0.5 µM; lane 3 1 µM; lanes 4–9 and 13 2 µM of purified *bc*PadR1 protein. Lanes 5 to 9 contains samples including increasing concentrations of AS-48 from 0.5 pM to 0.69 µM.

Genes associated with resistance mechanisms that are transcriptionally controlled by PadR-like proteins are generally encoded in the vicinity of the *padR* genes. Figure 1A shows the chromosomal regions around the two *B. cereus padR* genes, in comparison to the *lmrR-lmrCD* operon. The gene encoding *bc*PadR1 (locus BC4206 in *B. cereus* ATCC 14579) is located immediately downstream from BC4207, encoding a putative membrane protein of unknown function. These genes are predicted to share a common promoter, located immediately upstream from BC4206. Transcriptome analysis revealed that BC4206 and BC4207 are co-upregulated in response to enterocin AS-48 [14], indicating that these genes indeed form a bicistronic transcriptional unit. The same study also revealed that plasmid-based overexpression of BC4207 increased the resistance of *B. cereus* ATCC 14579 to AS-48, confirming the role of this gene in bacteriocin resistance, although its mechanism of operation is unknown. The BC4206-BC4207 transcriptional unit is conserved among *B. cereus* and *B. thuringiensis* species, but not present in the closely related *B. anthracis* [14]. To further examine the regulatory role of BC4206, we also overexpressed this gene in *B. cereus* ATCC 14579. Overexpression results in an increased sensitivity against AS-48 (MIC value of 1.0 µg/ml compared to a MIC value of 2.5 µg/ml for the wild-type strain, p-value<0.005, >6 cultures as determined with Student's t-test) suggesting that *bc*PadR1 acts as a repressor of the BC4206-BC4207 transcriptional unit, when AS-48 is not present in the medium. Electrophoretic mobility shift assays further reveal that purified *bc*PadR1 specifically binds to the intergenic region between BC4205 and the BC4206-BC4207 operon (Figure 1B and 1C). Incubation of the DNA probe with 0.5 µM *bc*PadR1 results in shifted bands, while higher *bc*PadR1 concentration results in stronger, but more diffused binding (Figure 1C). This might indicate the binding of two or more *bc*PadR1 dimers to the probe. The DNA probe used in these experiments contains the upstream regulatory region of BC4205 that codes for a putative spore photoproduct lyase. The expression of BC4205 is not altered in response to AS-48 addition [14], so its expression is likely to be unrelated to the AS-48 induced expression of BC4206-BC4207 operon. *In vitro* DNA binding of *bc*PadR1 was not affected by the addition of AS-48 suggesting an indirect sensing of AS-48 in the medium, although we cannot exclude the possibility that slightly altered *in vitro* conditions (e.g. different binding buffer) are needed to observe any effect of AS-48 addition on DNA binding.

The gene encoding *bc*PadR2 (locus BCE3449 in *B. cereus* ATCC 10987) lies downstream from two genes, BCE3447 and BCE3446, which encode a putative ABC-efflux pump. The product of BCE3447 forms the ATPase subunit of the transporter, while the product of BCE3446 forms the membrane permease. Interestingly, the amino acid sequence of the BCE3447-BCE3346 encoded pump is similar to that of the daunorubicin/doxorubicin resistance ABC transporter DrrAB from *Streptomyces peucetius* (overall sequence identity of 32%) [31], [32]. The sequence identity of the BCE3447-BCE3346 encoded pump with LmrCD is 27%, but LmrCD is about twice as large as BCE3447-BCE3446 and DrrAB. DNA analysis indicates that the BCE3449 and BCE3447-BCE3346 genes do not share a common promoter, and are separated by another gene, BCE3448, which encodes a soluble protein of unknown function. The structure of the BCE3448-encoded protein has been solved by a structural genomics consortium (PDB entry 2O3L) and shows a left-handed superhelix fold containing 5 α-helices. Genomic analysis reveals that the BCE3449-BCE3448-BCE3447-BCE3446 gene organization is conserved in various *Bacillus* species, but without functional expression data we cannot confirm that these genes form an operon or are coregulated.

### Sequence analysis

**Figure 2:**
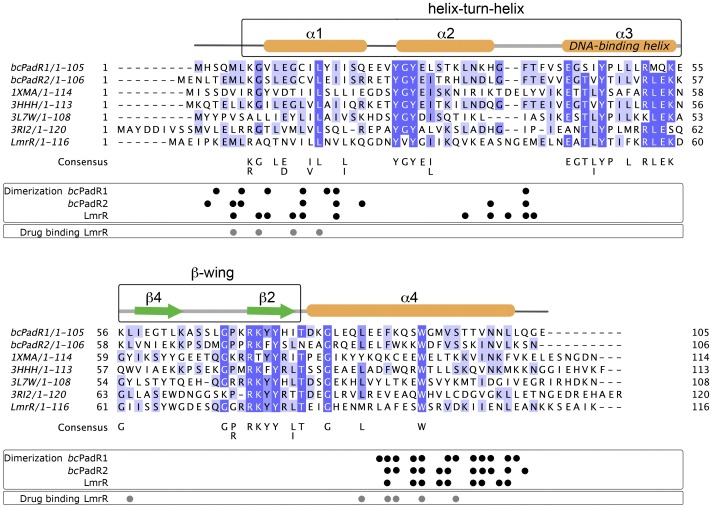
Multiple sequence alignment of *bc*PadR1, *bc*PadR2, and other members of the PadR-s2 subfamily. Only sequences for which structures are available in the PDB are shown in the alignment. The PadR-s2 proteins with unpublished structures are addressed by their PDB entry names: 1XMA, a putative transcriptional regulator from *Clostridium thermocellum*; 3HHH, a putative transcriptional regulator from *Enterococcus faecalis* V583; 3L7W, uncharacterized protein SMU.1704 from *Streptococcus mutans* UA159; 3RI2, a putative transcriptional regulator from *Eggerthella lenta* DSM 2243. Residues that participate in dimerization (for *bc*PadR1, *bc*PadR2, and LmrR) and/or have a role in drug binding (only for LmrR) are indicated by small spheres below the sequences. The consensus sequence is derived from a multiple sequence alignment of 2156 PadR-s2 proteins using as criteria that the conserved residue(s) should be present in at least 50% of the sequences.

*Bc*PadR1 and *bc*PadR2 contain 105 and 106 amino acid residues, respectively, and have all the characteristics of the PadR-s2 protein family. Figure 2 shows a multiple sequence alignment of the two proteins with other PadR-s2 proteins of which structures are currently deposited in the PDB, including LmrR. Pair-wise sequence identities range from 20% (*bc*PadR2 compared with a transcriptional regulator from *Clostridium thermocellum*, PDB entry 1XMA) to 61% (*bc*PadR2 compared with a transcriptional regulator from *Enterococcus faecalis* V583, PDB entry 3HHH). *Bc*PadR1 and *bc*PadR2 share a sequence identity of 45%, and are 26% and 30% identical in sequence to LmrR, respectively. Using an alignment-derived profile hidden Markov model, all homologs of *bc*PadR1 and *bc*PadR2 currently deposited in the Uniprot database were identified (>2150 sequences) and used to derive conserved sequence motifs. The highly conserved residues of the PadR-s2 proteins are mainly located in the wHTH domain and cluster in three regions, (i) at the N-terminus of helix α2 [motif YGYE(I/L)], (ii) in helix α3 [motif EGT(L/I)YPxLxRLE] and (iii) in the region encompassing strand β2 of the wing and the N-terminus of helix α2 [motif G(P/R)RKYYx(L/I)TxxG]. The identified sequence motifs partly overlap with the consensus sequence previously derived for all PadR-like proteins [4]. In addition to the above mentioned conserved sequence motifs, other highly conserved residues are found in more isolated positions at the N-terminus, helix α1 and the C-terminal helix of the subfamily 2 PadR-like proteins, including the central tryptophan residue in the C-terminal helix which has a central role in multidrug-binding in LmrR.

### Structure determination

To allow easy production of pure protein for crystallization, *bc*PadR1 and *bc*PadR2 were expressed with a C-terminal streptavidin-tag (SRWSHPQFEK) using an *L. lactis* nisin-overexpression system. Protein purification was carried out in two steps using Strep-Tactin and DNA affinity chromatography. Gel filtration experiments revealed that the recombinant *bc*PadR1 and *bc*PadR2 proteins have an apparent molecular weight of ∼43 kDa, similar as observed previously for LmrR and consistent with the presence of elongated dimers in solution (the calculated molecular weight of monomeric strep-tagged *bc*PadR1 and *bc*PadR2 is 13.3 kDa and 13.7 kDa, respectively)

*Bc*PadR1 was crystallized in space group *P*4_1_2_1_2, with one protein molecule per asymmetric unit and a solvent content of 71%. Similarly, *bc*PadR2 was crystallized in space group *P*4_3_2_1_2, with one protein molecule per asymmetric unit and a solvent content of 42%. The *bc*PadR structures were determined by molecular replacement using the structure of the PadR-like protein from *E. faecalis* V583 as a search model. Model building and refinement were somewhat hampered by relatively high atomic displacement parameters (B-factors), indicative of some global disorder or flexibility of the proteins, even though the overall fit to the electron density is well defined. The final refined structures of *bc*PadR1 and *bc*PadR2 have an R-factor/R_free_ of 20.2/22.6% at 2.5 Å resolution and 22.3/27.7% at 2.2 Å resolution, respectively, with good geometry (see Table 1 for details of the refinement statistics). The two structures are highly similar with a Cα-backbone root mean square deviation (RMSD) of 1.45 Å (for 96 common residues). The N-termini (residues 1–2 in both *bc*PadR proteins) and the C-terminal strep-tags (residues 106–115 in *bc*PadR1 and residues 108–116 in *bc*PadR2) were not included in the models due to weak or absent electron density. In addition, residues 67–71 in the β-wing of *bc*PadR2 were found to be disordered and therefore left out from the model.

### Overall structures of bcPadR1 and bcPadR2

**Figure 3:**
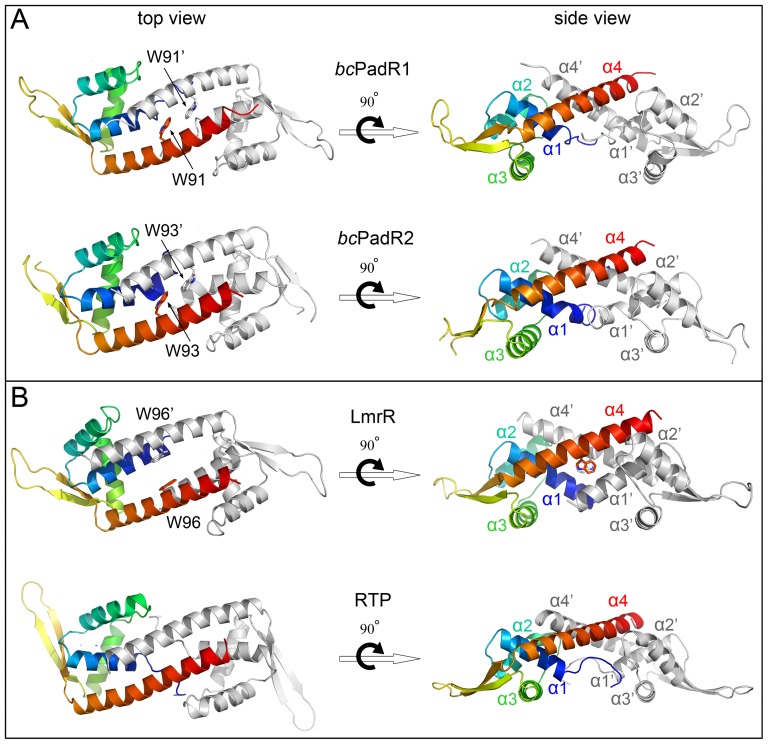
Ribbon representations of the *bc*PadR dimers and selected structural homolog. (A) *bc*PadR1, (B) *bc*PadR2, (C) LmrR (PDB code 3F8B), and (D) RTP, a replication terminator protein from *Bacillus subtilis* (PDB code 1F4K). For each dimer, one of the subunits is shown in a rainbow color gradient from the N-terminus (blue) to the C-terminus (red), whereas the other subunit is colored grey. Helices involved in dimerization are indicated. Residues Trp91 of *bc*PadR1, Trp93 of *bc*PadR2, and Trp96 of LmrR are drawn in stick representation.

In the crystals, *bc*PadR1 and *bc*PadR2 form similar dimers, with a dimeric 2-fold axis that coincides with a crystallographic 2-fold axis. The approximate dimensions of the dimers are 80 Å×34 Å×30 Å (Figure 3A). The *bc*PadR1 and *bc*PadR2 subunits contain a typical wHTH DNA-binding domain, consisting of helices α1, α2, the DNA recognition helix α3 and strands β1 and β2 (together forming the wing), and a dimerization domain, including the C-terminal helix α4. Helix α4 forms a protruding arm which in the dimer interacts with helices α4′ and α1′ of the dimer-mate. Dimerization of *bc*PadR1 and *bc*PadR2 buries approximately 1740 Å^2^ solvent-accesible surface area per subunit which is related to ∼30% of the total surface area. The dimeric interface involves mainly van der Waals contacts between hydrophobic residues, most of which are conserved between the two *bc*PadR proteins (see Figure 2). In addition a few hydrogen bonds are observed at the dimer interface, but these are not conserved.

### BcPadR1 and bcPadR2 show a closed dimeric interface

**Figure 4:**
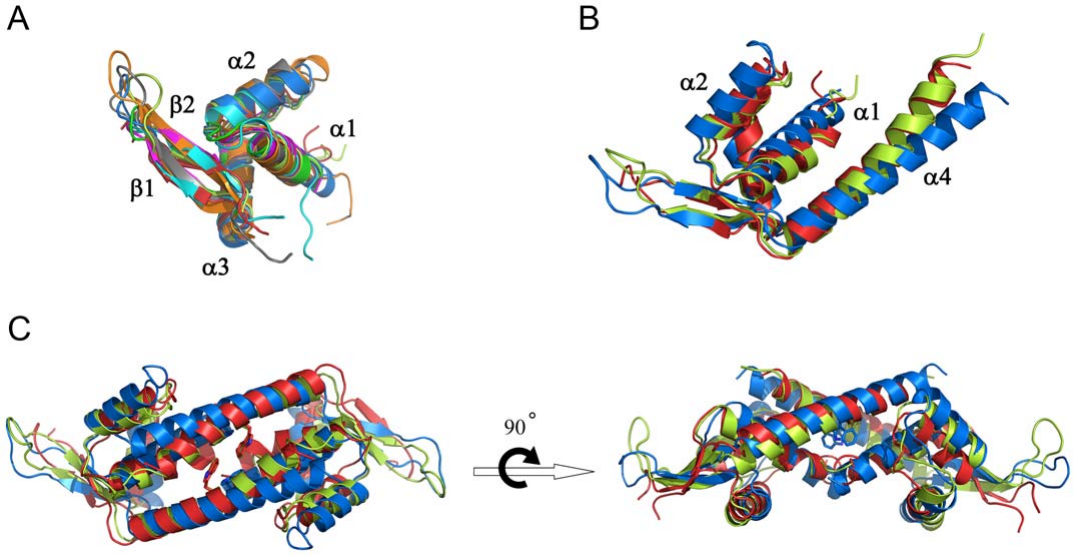
Structural comparison between *bc*PadR1, *bc*PadR2 and LmrR. (A) Superpositions of the wHTH domains of *bc*PadR1 (light-green), *bc*PadR2 (red), LmrR (blue), and the following homologs: MexR (green), SmtB (magenta), BlaI (cyan), RTP (orange), and Pex (gray) with the secondary structure elements indicated and labeled. (B) Superposition of the single *bc*PadR1, *bc*PadR2 and LmR subunits. (D) Superposition of the *bc*PadR1, *bc*PadR2 and LmR dimers.

Although similar in overall fold, it is evident that the two *bc*PadR structures do not show the same dimeric interface as LmrR (Figures 3 and 4). Unlike LmrR, but like the other PadR-s2 proteins with known structure, the *bc*PadR1 and *bc*PadR2 dimers have a completely closed dimer interface. The differences at the dimer interface are associated with changes in the relative orientation of helices α1 and α4 with respect to the core of the wHTH domain (Figure 4B). In addition, helix α4 in *bc*PadR1 and *bc*PadR2 shows significant bending towards the dimeric centre, whereas the equivalent helix in LmrR is almost perfectly straight (Figure 4C). As a consequence, helices α4 and α4′ in the *bc*PadR dimers can approach each other closely at the dimer interface, allowing a coiled-coil interaction mode. In LmrR the C-terminal helices do not make any mutual contacts, but, instead, interact strongly with helices α1 and α2 of the dimer mate. Intriguingly, despite these significant differences in dimeric organization, the residues in *bc*PadR proteins and LmrR that form the inter-subunit interactions are located at similar positions in the structures (Figure 2). It is unclear how the differences in amino acid sequence at these regions affect the conformation of the polypeptides in the dimer. In particular, the highly conserved tryptophan residues in the C-terminal helices of the *bc*PadR dimers (Trp91/Trp91′ and Trp93/Trp93′ in *bc*PadR1 and *bc*PadR2, respectively), which are the equivalent of Trp96/Trp96′ in LmrR, are completely buried in the dimer interface. The closed dimer conformation and inaccessibility of the conserved tryptophan residues of the *bc*PadR proteins are incompatible with a drug-binding mechanism as observed in LmrR.

### Structural similarity with non-PadR-like transcription factors

A structural similarity search against available structures in the Protein Data Bank (PDB) using the DALI server [33] reveals that *bc*PadR1 and *bc*PadR2 are not only similar to other PadR-like proteins, but also to various non-PadR-like transcription factors including members of the MarR family, SmtB/ArsR family and the MecI/BlaI family (Table 2). These proteins all contain wHTH-domains for DNA binding and form functional dimers. The similarities of the non-PadR-like structural homologs of *bc*PadR1 and *bc*PadR2 are mainly limited to the wHTH domains, which can be superimposed to those of *bc*PadR1 and *bc*PadR2 with RMSDs (for Cα-backbone superpositions) of 1.1–1.9 Å (Figure 4A). In contrast, the dimerization topologies of the non-PadR-like structural homologs of *bc*PadR1 and *bc*PadR2 are highly diverse, often including additional helices at the N- and C-terminal regions. One notable exception is the transcription factor RTP (replication terminator protein) from *Bacillus subtilis*, which plays a crucial role in the coordinated termination of DNA replication [34]. Despite the absence of significant sequence homology, *bc*PadR1 and *bc*PadR2 show a highly similar overall topology and dimeric organization as RTP, including the presence of a single C-terminal helix for dimerization (Figure 3). The striking similarities of the *bc*PadR and RTP structures strongly suggest that these proteins use a common mode of DNA binding.

**Table 2:** DALI-identified structural homologs of *bc*PadR1 and *bc*PadR2.*

	*bc*PadR1/*bc*PadR2							
PDB-ID	Z-score	RMSD (Å)	Cα aligned	Nr. res	% id.	Protein name	Protein family	Ref.
3HHH	16.1/17.7	1.6/1.7	101/101	103/103	42/61	n.k.	PadR-s2	u.
3L7W	14.2/13.6	2.0/2.7	99/100	106/106	27/27	n.k.	PadR-s2	u.
3RI2	14.0/14.0	2.5/1.6	99/100	112/112	30/26	n.k.	PadR-s2	u.
1XMA	13.9/14.8	2.6/1.5	97/98	103/101	22/20	n.k.	PadR-s2	u.
1LNW	12.8/12.0	2.7/3.0	93/88	141/141	13/16	MexR	MarR	[40]
3F8B	12.5/11.7	3.1/2.7	100/98	105/101	23/30	LmrR	PadR-s2	[9]
1YG2	11.0/10.6	3.2/2.8	83/82	169/169	22/24	AphA	PadR-s1	[8]
1F4K	10.6/11.0	3.1/2.5	98/98	115/115	12/15	RTP	RTP	[34]
1SMT	10.1/9.5	2.6/2.9	78/77	98/101	14/19	SmtB	SmtB/ArsR	[41]
1SD6	9.7/9.6	3.3/3.1	84/84	119/119	15/19	BlaI	MecI/BlaI	[35]
2E1N	9.3/9.1	3.5/3.1	90/89	112/112	21/25	Pex	PadR-s1	[7]

Abbreviations: PadR-s1, PadR-like subfamily-1; PadR-s2, PadR-like subfamily-2, n.k., not known; u, unpublished.

* The DALI-search was performed using a single subunit of each *bc*PadR dimer.

### Putative DNA-binding interactions

**Figure 5:**
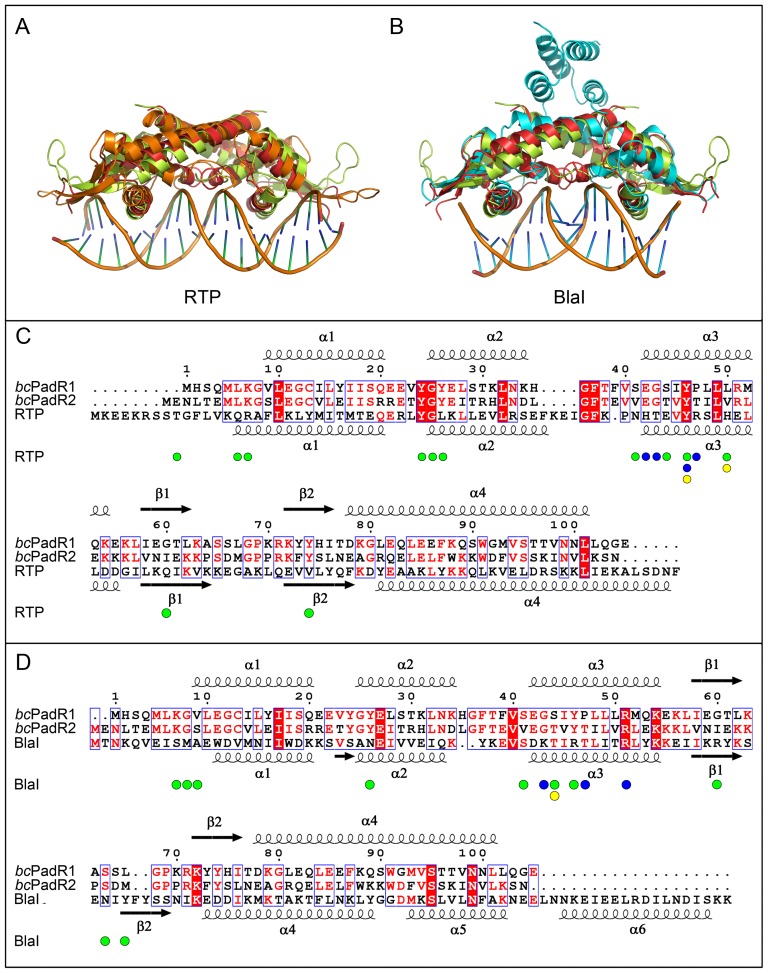
Modeling of the DNA-bound *bc*PadR complexes. (A) Superposition of the *bc*PadR1 (light-green) and *bc*PadR2 (red) dimers onto DNA-bound RTP (orange, PDB code 1F4K). (B) Superposition of the *bc*PadR1 (light-green) and *bc*PadR2 (red) dimers onto DNA-bound BlaI (cyan, PDB code 1XSD). Below the superpositions are structure-based sequence alignments including secondary structures. Residues of RTP and BlaI which participate in DNA binding are indicated with small spheres using the following color scheme: green, interacting with the phosphate backbone; blue, interacting with the base moiety and yellow, interacting with the ribose backbone).

To understand how *bc*PadR1 and *bc*PadR2 may bind to DNA, the *bc*PadR structures were superimposed to structures of close homologs in complex with DNA (Figure 5). At present no structure is available of a PadR-like protein bound to its cognate DNA. However, DNA-bound structures are available for two of the non-PadR-like close homologs identified by the DALI search, i.e., the replication terminator protein RTP (PDB entry 1F4K) and the transcriptional repressor BlaI (PDB entry 1XSD) [34], [35]. RTP and BlaI exploit common principles to fulfill their role as DNA-binding regulatory proteins, which most probably are also used by the *bc*PadR proteins. The operator sequences of RTP and BlaI contain specific inverted repeat motifs and the proteins bind such that their dimeric dyads are aligned with the inverted repeat dyads of the DNA operators. In this way, the recognition helices (α3) of the dimer-related wHTH domains can insert into two successive major grooves of the DNA, while the β-wings lie across the phosphate backbone, within the vicinity of adjacent minor groove clefts. DNA binding is stabilized by electrostatic interactions between the highly positively charged surface of the DNA binding site and the negatively charged DNA backbone. Additional DNA:protein interactions comprise various non-bonding contacts, including hydrophobic interactions, and a few hydrogen bond interactions. Residues in the α3-helix are responsible for specific DNA binding and recognition of the operator DNA sequence, by penetrating into the major groove and forming hydrogen bonds with bases of the nucleotides.

**Figure 6:**
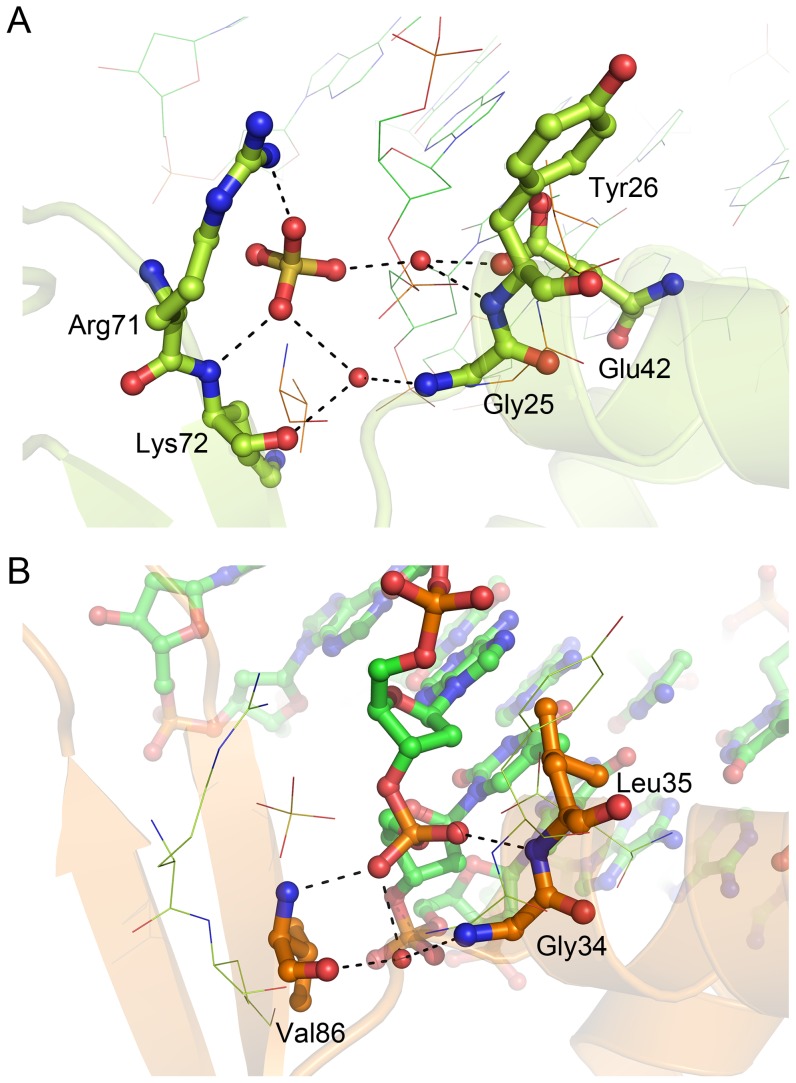
The bound sulfate anion in the *bc*PadR1 structure. (A) Interactions between *bc*PadR1 residues and the sulfate ion. Also shown is the DNA-bound RTP structure (PDB entry 1F4K) onto which the *bc*PadR1 dimer was superimposed. The *bc*PadR1 residues and the sulfate ion are shown in ball-and-stick representation and labeled, while the DNA strand of the RTP-DNA complex is drawn using lines. (B) Interactions between the RTP residues and the DNA phosphate backbone adjacent to the sulfate anion in the superimposed *bc*PadR1 structure. The RTP residues and DNA strands are shown in ball-and-stick representation and labeled, while the *bc*PadR1 residues and sulfate are drawn using lines.

The *bc*PadR1 dimer can be superimposed to the DNA-bound complexes of RTP and BlaI with RMSD values of 3.5 Å (for 176 common Cα atoms divided equally between both subunits) and 4.1 Å (for 153 common Cα atoms), respectively. Likewise, the *bc*PadR2 dimer can be fitted to the DNA-bound complexes of RTP and BlaI with RMSD values of 2.9 Å (for 179 common Cα atoms) and 3.4 Å (for 155 common Cα atoms). Overall, the dimers of *bc*PadR1 and *bc*PadR2 are highly similar to RTP and BlaI, but there are small differences in the relative dispositions of the dimer-related subunits which affect the relative orientations of the α3 helices. Since DNA-binding by transcription factors is often associated with specific conformational changes in both protein and DNA, the superpositions cannot be used to accurately predict the details of the DNA binding mechanism of *bc*PadR1 and *bc*PadR2. However, analysis of the models in combination with a structure-based sequence alignment of *bc*PadR1, *bc*PadR2, RTP and BlaI (Figure 5) leads to a few interesting observations. First, like in RTP and BlaI, the complementary surfaces in *bc*PadR1 and *bc*PadR2 that form the putative DNA binding sites are highly positively charged, due to the presence of various lysine and arginine residues. A number of these lysine and arginine residues from part of the previously described conserved sequence motifs in the PadR-s2 proteins, in particular those located in helix α3 and the β-wing, strongly implying a direct role in DNA binding. Furthermore, the conserved YGYE motif in *bc*PadR1 and *bc*PadR2, which is located at the N-terminal end of helix α2, is partly preserved in RTP (residues 33–36 with sequence YGLK). In RTP, the side chain of Tyr33, together with the backbone amides of Gly34 and Leu35, forms hydrogen bonds with the phosphate-backbone of the DNA. As these interactions are crucial for the strong DNA binding affinity of RTP [36], it is likely that the conserved residues in *bc*PadR1 and *bc*PadR2 have a similar DNA binding role. This is corroborated by the observation of a bound sulfate anion in the *bc*PadR1 structure, which forms interactions with the highly conserved residues Gly25, Tyr26, Glu42 Arg71, and Lys72, likely mimicking the interactions with a phosphate moiety of the DNA backbone (Figure 6). Finally, the α3 helices of the *bc*PadR proteins show a limited number of conserved residues relative to the equivalent helices in RTP and BlaI. Interestingly, two residues of the conserved EGTLYPxLxRLE motif are identical to sequence-specific DNA binding residues in RTP or BlaI, i.e., Tyr46 and Arg51, strongly suggesting that these residues in helix α3 of the *bc*PadR proteins have a similar DNA binding role. However, the limited overall sequence conservation of the α3 helices is also indicative of the differences that must exist between the specific cognate operator sequences of these DNA-binding proteins.

## Discussion

In this study, we have determined the crystal structures of *bc*PadR1 and *bc*PadR2, which are the products of gene loci BC4206 and BCE3449 of *B. cereu*s strains ATCC 14579 and ATCC 10987, respectively. Functional data point toward the hypothesis that *bc*PadR1 autoregulates the transcription of its own gene, as *bc*PadR1 binds to the promoter region of gene BC4206 and overexpression of BC4206 gene results in increased sensitivity against AS-48. The structures of *bc*PadR1 and *bc*PadR2 are similar to LmrR, containing a conserved N-terminal wHTH DNA binding domain and a C-terminal helix involved in dimerization. The conserved structures and sequence relationships confirm that all three proteins belong to the same subfamily of small PadR proteins (subfamily 2 according to Huillet et al. [4], which we abbreviated as PadR-s2.) The identification of conserved sequence motifs in the wHTH domains, in particular in the α3 DNA recognition helix, further suggests that the PadR-s2 proteins share a common cognate DNA operator sequence. Operator sites of LmrR in the *lmrR* and *lmrCD* promoter regions have been identified previously [5], [12], and were shown to contain an ATGT/ACAT inverted sequence motif, separated by a short spacer of about 8–10 nucleotides. The inverted repeat is identical to the conserved DNA sequence motif revealed for members of the other subfamily of PadR proteins (PadR-s1 family) [1], [37]. Indeed, similar sequence motifs, although somewhat degenerate, are present in the promoter of the BC4206–4207 operon [ATGT-(N)_10_-ATAA, Figure 1B] and the putative promoter region preceding BCE3449 [CTGT-(N)_8_-ATAT, data not shown]. Functional studies and footprinting analysis are required to fully establish the identity of the target DNA operator sites and to identify other possible target genes controlled by *bc*PadR1 and *bc*PadR2.

Compared to related transcription factors of the MarR, SmtB/ArsR and MecI/BlaI families, the *bc*PadR proteins show a “minimal” fold, lacking extra N- and C-terminal helices associated with dimerization. In the non-PadR-like proteins the extra helices often play a role in the mechanisms of transcriptional induction, allowing modulation of DNA binding activities through direct binding of a ligand. Also the structural features in LmrR that are related with its role as a multi-drug induced transcription regulator, i.e., a large central pore with two opposing tryptophan residues for multi-drug binding, are notably absent in *bc*PadR1 and *bc*PadR2. These observations suggestthat the DNA binding activities of *bc*PadR1 and *bc*PadR2 are not directly modulated by ligands. In line with this hypothesis, *in vitro* DNA binding of *bc*PadR1 was not altered by the presence of AS-48. This is not surprising since AS-48 is known to target the cell membrane, inducing membrane pore formation [14], and is unlikely to enter the cytoplasm. Perhaps, the *bc*PadR proteins undergo a conformational change, which opens the dimer interface, thereby forming a ligand binding site. Alternatively, transcriptional regulating by the *bc*PadR proteins may involve an indirect induction mechanism with the participation of another protein. Such a mechanism has been proposed for the phenolic acid stress response in *L. plantarum*, which is regulated by the founding member of the PadR-like transcription factors and involves a protein belonging to the universal stress protein family [38], [39]. Examining such possibilities for the mechanism of action of the *bc*PadR proteins has to await experimental data characterizing their regulatory functions and identifying the target genes which they control.
